# Комплексный анализ постковидных кардиоренальных осложнений у пациентов с сахарным диабетом 1 и 2 типа по данным мобильного лечебно-диагностического центра «Диамобиль»

**DOI:** 10.14341/probl13426

**Published:** 2024-09-15

**Authors:** О. К. Викулова, А. В. Железнякова, А. А. Серков, М. А. Исаков, Г. Р. Вагапова, Ф. В. Валеева, Н. П. Трубицына, О. Г. Мельникова, В. К. Александрова, Н. Б. Смирнова, Д. Н. Егорова, Е. В. Артемова, К. В. Сорокина, М. В. Шестакова, Н. Г. Мокрышева, И. И. Дедов

**Affiliations:** Национальный медицинский исследовательский центр эндокринологии; Национальный медицинский исследовательский центр эндокринологии; Национальный медицинский исследовательский центр эндокринологии; Национальный медицинский исследовательский центр эндокринологии; Казанская государственная медицинская академия; Казанский государственный медицинский университет; Национальный медицинский исследовательский центр эндокринологии; Национальный медицинский исследовательский центр эндокринологии; Национальный медицинский исследовательский центр эндокринологии; Национальный медицинский исследовательский центр эндокринологии; Национальный медицинский исследовательский центр эндокринологии; Национальный медицинский исследовательский центр эндокринологии; Национальный медицинский исследовательский центр эндокринологии; Национальный медицинский исследовательский центр эндокринологии; Национальный медицинский исследовательский центр эндокринологии; Национальный медицинский исследовательский центр эндокринологии

**Keywords:** сахарный диабет 2 типа, сахарный диабет 1 типа, постковидный синдром, мобильный медицинский центр «Диамобиль», кардиоренальные осложнения

## Abstract

**РЕЗЮМЕ:**

РЕЗЮМЕ. Пациенты с сахарным диабетом (СД) относятся к группе риска большей частоты и тяжести течения COVID-19, а также его неблагоприятных исходов, в том числе постковидного синдрома.

**ЦЕЛЬ:**

ЦЕЛЬ. Оценить частоту развития кардиоренальных осложнений у пациентов с СД 1 и 2 типа, перенесших COVID-19, и провести анализ структуры и тяжести нарушений по данным обследования в мобильном медицинском лечебно-диагностическом центре «Диамобиль».

**МАТЕРИАЛЫ И МЕТОДЫ:**

МАТЕРИАЛЫ И МЕТОДЫ. Когорта пациентов с СД 1 и 2 типа, обследованных в «Диамобиле» (n=318), с подтвержденным в анамнезе COVID-19 (n=236). Временной интервал между COVID-19 и визитом в «Диамобиль» составил 8,7/8,2 мес. при СД1/СД2. В качестве исходных данных использовались параметры последнего визита до COVID-19, зафиксированного в Федеральном регистре СД (ФРСД).

**РЕЗУЛЬТАТЫ:**

РЕЗУЛЬТАТЫ. Клиническая характеристика пациентов с СД1/СД2: возраст — 49,2/64,5 года, длительность СД — 22/11 лет, доля женщин — 64/73% соответственно. При анализе данных визитов до и после COVID-19 статистически значимых отличий по уровню HbA1c при обоих типах СД не отмечено (до — 9,0/8,3%; после — 8,4/8,2% соответственно), в том числе вследствие интенсификации терапии (возросла доля пациентов с СД2 на 2- и 3-компонентной терапии на 4,3 и 1,6%, доля пациентов на инсулинотерапии — на 16%). После COVID-19 отмечалось статистически значимое снижение скорости клубочковой фильтрации (СКФ) при СД1 с 88,1 до 62 мл/мин/1,73 м2; при СД2 — с 74,7 до 54,1 мл/мин/1,73 м2. При оценке острых диабетических осложнений отмечено увеличение частоты ком при СД1 в 1,5 раза, тяжелых гипогликемий при СД1 — в 3 раза, при СД2 — в 1,7 раза. Анализ частоты кардиоренальных осложнений до и после COVID-19 показал суммарное увеличение на 8,5% — при СД1, на 13,2% — при СД2, из них инфаркт миокарда, ИБС, ХСН увеличились при СД1 в диапазоне от 1,5 до 5 раз, при СД2 — в 1,3 раза, частота ХБП при СД1 — в 1,5 раза, при СД2 — в 5,6 раза.

**ВЫВОДЫ:**

ВЫВОДЫ. При стабильных показателях HbA1c, достигнутых на фоне интенсификации терапии в период COVID-19, в постковидном периоде отмечается ухудшение функциональной способности почек (снижение СКФ) и увеличение частоты сердечно-сосудистых осложнений при обоих типах СД, что отражает факт сочетанного поражения почек и сердечно-сосудистой системы в рамках постковидного синдрома и определяет ключевой спектр мероприятий для разработки мер профилактики.

## Abstract (en)

**BACKGROUND:**

BACKGROUND: Patients with diabetes mellitus (DM) are at risk for a higher incidence and severity of COVID-19, as well as its adverse outcomes, including post-Covid syndrome.

**AIM:**

AIM: to assess the incidence of cardiorenal complications in patients with type 1 and type 2 diabetes (T1DM/T2DM) who have had COVID-19, and to analyze the structure and severity of disorders according to examination data at the Diamobil mobile medical diagnostic and treatment center.

**MATERIALS AND METHODS:**

MATERIALS AND METHODS: a cohort of T1DM and T2DM patients examined in Diamobil (n=318), with a confirmed anamnesis of COVID-19 (n=236). The time interval between COVID-19 and the visit to Diamobil was 8.7/8.2 months for T1DM/T2DM. The parameters of the last visit before COVID-19 recorded in the Federal Register of Diabetes (FRD) were used as initial data.

**RESULTS:**

RESULTS: Clinical characteristics of patients with T1DM/T2DM: age — 49.2/64.5 years, duration of DM — 22/11 years, proportion of women — 64/73%, respectively. After analysis the data from visits before and after COVID-19 there weren’t statistically significant differences in HbA1c levels for both types of DM (before 9.0/8.3%; after 8.4/8.2%, respectively), there was the intensification of glucose lowering therapy (the proportion of patients with T2DM on 2 and 3 component therapy increased by 4.3% and 1.6%, the proportion of patients on insulin therapy by 16%). After COVID-19, there was a statistically significant decrease in glomerular filtration rate (GFR) in T1DM from 88.1 to 62 ml/min/1.73 m2; with T2DM from 74.7 to 54.1 ml/min/1.73 m2. When assessing acute diabetic complications, there was an increase in the frequency of coma in T1DM by 1.5 times, severe hypoglycemia in T1DM by 3 times, and in T2DM by 1.7 times. Analysis of the frequency of cardiorenal complications before and after COVID-19 showed a total increase of 8.5% in T1DM, by 13.2% in T2DM, of which myocardial infarction, ischemic heart disease, and CHF increased in T1DM in the range from 1.5 to 5 times, with T2DM by 1.3 times, the frequency of CKD with T1DM by 1.5 times, with T2DM by 5.6 times.

**CONCLUSION:**

CONCLUSION: There was a decline of kidney filtration function (decrease in GFR) and an increase in the frequency of cardiovascular complications in both types of diabetes in post-Covid period while patients achieved a stable HbA1c levels by intensifying therapy during the COVID-19 infection. This fact reflects combined damage to the kidney and cardiovascular system as a part of the post-Covid syndrome and determines a key set of measures for the development of preventive strategies.

## ОБОСНОВАНИЕ

Пандемия новой коронавирусной инфекции (COronaVIrusDisease — 2019, COVID-19) стала глобальным вызовом национальным системам здравоохранения во всех странах мира вследствие повышения смертности, поставив новые задачи для организации медицинской помощи. По данным Всемирной организации здравоохранения (ВОЗ), по состоянию на 8 ноября 2023 г. во всем мире зарегистрировано 771 820 937 подтвержденных случаев заболевания COVID-19, в том числе 6 978 175 случаев смерти, что составляет 0,9% летальности [1]. По данным статистики, предоставленной Роспотребнадзором, в Российской Федерации (РФ) на 16 ноября 2023 г. было заражено 23 014 969 человек, из которых 22 458 308 выздоровели и 400 023 скончались, что составляет 1,74% летальности [2].

COVID-19 стал своеобразной моделью изучения сердечно-сосудистых рисков у различных категорий пациентов, в том числе у пациентов с сахарным диабетом (СД). Продолжается изучение патогенеза COVID-19, оптимизации аспектов ведения постковидного синдрома и обсуждение дальнейшего наблюдения пациентов, после перенесенной инфекции. В настоящий момент ни у кого не вызывает сомнений, что наиболее тяжелое течение COVID-19 наблюдается у групп риска с хроническими заболеваниями, к которым относятся пациенты с СД.

Высокая распространенность как COVID-19, так и СД, а также патогенетические особенности инфекции определяют сочетание этих заболеваний как предпосылку развития наиболее неблагоприятного прогноза. По данным эпидемиологических исследований, СД является второй по распространенности сопутствующей патологией при COVID-19 после сердечно-сосудистых заболеваний (ССЗ) [3]. По данным ГНЦ ФГБУ «НМИЦ эндокринологии» Минздрава России, летальность вследствие COVID-19 среди пациентов с СД в 5–8 раз выше общепопуляционных показателей: при СД1 — 7–8% и 18% при СД2 [4][5]. Таким образом, пациенты с СД относятся к одной из наиболее уязвимых групп риска при COVID-19, более тяжелого течения инфекции, повышения риска неблагоприятных исходов, в том числе смертности, а также развития постковидного синдрома.

Риски связаны не только с острой фазой заболевания, но и с различными последствиями перенесенной инфекции, которые в настоящее время получили название постковидного синдрома [6]. Для классификации и учета постковидных нарушений был введен код U009 в Международный классификатор болезней Десятого пересмотра (МКБ-10) с формулировкой «Post-COVID-19» «Состояние после COVID-19», который объединяет учет симптомов, развивающихся после COVID-19, «необъяснимых альтернативным диагнозом» [7][8].

В настоящее время постковидный синдром рассматривается как полисиндромальное состояние с поражением различных органов и систем [9]. Сведения по персонализации рисков и частоты постковидного синдрома в когорте пациентов с СД ограничены.

В связи с этим анализ частоты развития кардиоренальных осложнений в рамках постковидного синдрома в когорте пациентов с СД представляет особую актуальность с целью адресной оценки ситуации на региональном уровне и повышения качества диабетологической помощи в субъектах РФ. Начиная с 2002 г. ГНЦ ФГБУ «НМИЦ эндокринологии» Минздрава России осуществляет системный клинико-эпидемиологический мониторинг СД в РФ посредством ежегодных эпидемиологических выездов в регионы мобильного медицинского лечебно-диагностического центра «Диамобиль» [10][11]. Данная модель обследования пациентов оптимальна для своевременной диагностики поражения органов-мишеней при СД и с 2022 г. стала использоваться для оценки постковидного синдрома в условиях «Диамобиля».

Цель исследования: выполнить комплексный динамический мониторинг кардиоренальных осложнений в рамках постковидных нарушений у пациентов с СД1 и СД2, перенесших COVID-19, с анализом их структуры и тяжести посредством обследования в мобильном медицинском лечебно-диагностическом центре «Диамобиль».

## МАТЕРИАЛЫ И МЕТОДЫ

Настоящая статья посвящена оценке распространенности кардиоренальных осложнений в рамках постковидных нарушений у пациентов с СД1 и СД2, перенесших COVID-19.

## Место проведения

Выезд мобильного медицинского лечебно-диагностического центра «Диамобиль» в Республику Татарстан.

## Время исследования

Выезд «Диамобиля» состоялся 16–27 мая 2022 г.

Изучаемая популяция: пациенты с СД1 и СД2, перенесшие COVID-19 в период пандемии с 01.2020 по 03.2022 гг. Всего обследовано 318 пациентов, из них с СД1 (n=95) и СД2 (n=220), из которых подтвержденный COVID-19 зафиксирован у 74,2% пациентов (n=236; СД1 — 50, СД2 — 186).

Дизайн исследования: одноцентровое, скрининговое исследование с ретроспективным анализом данных.

Медиана временного интервала от COVID-19 до обследования в «Диамобиле» была рассчитана в месяцах по количеству дней между датами начала заболевания и осмотра в «Диамобиле» по формуле: N(дней)/30 (рис. 1).

**Figure:**
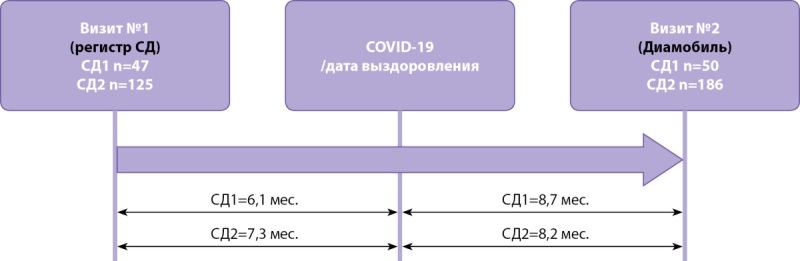
Рисунок 1. Временные интервалы между визитами пациентов до и после COVID-19.

Для оценки динамики клинического статуса пациентов до и после перенесенного COVID-19 в качестве исходных данных использовались параметры последнего предшествующего развитию инфекционного заболевания визита к эндокринологу, зафиксированного по данным регионального сегмента регистра СД (рис. 1).

Медиана временных интервалов до COVID-19 (между исходным визитом в регистре и началом заболевания) составила при СД1 6,1 мес. [ 3,6; 9,2], при СД2 — 7,3 [ 3,9; 13,3] мес.; после COVID-19 — для СД1 8,7 мес. [ 5,1; 13,4], для СД2 — 8,2 мес. [ 5,0; 14,5] (рис. 1).

В итоговый анализ включены 172 пациента (СД1 — 47, с СД2 — 125), у которых имелись данные обоих визитов.

## Методы

Общий объем исследований в мобильном медицинском лечебно-диагностическом центре «Диамобиль» соответствует стандарту обследования, направленного на оценку контроля углеводного обмена и состояния всех органов-мишеней при СД, раннюю диагностику диабетических осложнений согласно «Алгоритмам специализированной медицинской помощи больным сахарным диабетом» [12].

Обследование включало: оценку антропометрических данных (рост, вес, ИМТ), биохимическое исследование липидного спектра крови и уровня креатинина с расчетом скорости клубочковой фильтрации (СКФ-EPI), альбуминурии (АУ) и соотношения альбумин/креатинин (А/Кр) в разовой порции мочи, уровня гликированного гемоглобина (HbA1c), артериального давления (АД), ЭКГ, консультацию кардиолога, офтальмолога, специалиста кабинета «Диабетическая стопа», диабетолога. Исследование всех биохимических показателей выполнялось с помощью коммерческих наборов на биохимическом экспресс-анализаторе Spotchem EZ ArkraySP-4430. Определение HbA1c, АУ и А/Кр выполнялось иммунохимическим методом на анализаторе DCA Vantage. Размер выборки предварительно не рассчитывался. Все пациенты подписали добровольное информированное согласие на участие в обследовании в «Диамобиле».

## Статистический анализ

Анализ сформированных данных проводился с помощью пакета прикладных программ Statistica v.13.3 (TIBCO Software Inc., США). Описательная статистика количественных признаков представлена в виде медиан и квартилей [Q1; Q3], для качественных — в виде абсолютных и относительных частот n (%), N — количество пациентов. Динамическое сравнение количественных параметров в группах наблюдения до и после перенесенного COVID-19 выполнялось с помощью критерия Вилкоксона, качественных — с помощью критерия Мак-Немара. Сравнительный анализ независимых групп по качественным признакам выполнялся с помощью критерия Хи-квадрат (х²). Критический уровень статистической значимости при проверке статистических гипотез принят равным 0,05.

## Этическая экспертиза

Протокол исследования был одобрен локальным этическим комитетом Эндокринологического научного центра, Москва, Россия, 30 апреля 2020 г., протокол №6.

## РЕЗУЛЬТАТЫ

Клиническая характеристика пациентов с СД1 и СД2 с подтвержденным COVID-19, по данным обследования в «Диамобиле», представлена в таблице 1. Пациенты с СД1 были средней возрастной категории (49,2 года), с длительным анамнезом СД (22 года), доля женщин составила 64%, медиана HbA1c — 8,4%. Пациенты с СД2 были старшей возрастной категории (64,5 года), медиана длительности диагноза СД — 11 лет, основную долю составляли женщины (73%), медиана уровня HbA1c была 8,1%.

**Table:** Таблица 1. Клиническая характеристика перенесших COVID-19 пациентов с СД1 и СД2 на момент обследования в «Диамобиле», Республика Татарстан 2022 г. (n=236) Данные представлены в процентах, %, медианой и первым, третьим квартилями (Mediana [ Q1; Q3]). АД представлено в виде средних значений. HbA1c — гликированный гемоглобин; АД — артериальное давление; ИМТ — индекс массы тела; СКФ — скорость клубочковой фильтрации; ЛПВП — липопротеины высокой плотности; ЛПНП — липопротеины низкой плотности.

Параметр	СД1(n=50)	СД2(n=186)
Пол, ж/м, %	64/36	73,1/26,9
Текущий возраст, лет	49,2 [ 39; 61]	64,5 [ 60; 70]
Длительность СД, лет	22 [ 13; 32]	11 [ 4; 18]
HbA1c, %	8,4 [ 7,3; 9,3]	8,1 [ 6,9; 9,2]
Диастолическое АД, мм рт.ст.	80 [ 70; 80]	80 [ 75; 90]
Систолическое АД, мм рт.ст.	125 [ 120; 140]	145 [ 130; 155]
ИМТ, кг/м²	25,8 [ 23,1; 28,1]	32,5 [ 29,1; 36]
Холестерин, ммоль/л	4,7 [ 4,1; 5,2]	4,8 [ 4,0; 5,5]
ЛПВП, ммоль/л	2,2 [ 1,7; 2,7]	1,4 [ 1,2; 1,7]
ЛПНП, ммоль/л	1,9 [ 1,5; 2,9]	2,4 [ 1,8; 3]
Триглицериды, ммоль/л	0,6 [ 0,3; 0,8]	1,5 [ 1,1; 2,5]
Альбумин/креатинин, мг/ммоль	0,9 [ 0,6; 1,9]	1,45 [ 0,9; 2,4]
СКФ (CKD-EPI), мл/мин/1,73м²	64,8 [ 53,4; 77,2]	53,5 [ 46,4; 61,2]

Динамика лабораторных показателей до и после COVID-19 в «Диамобиле» представлена в таблице 2. Анализ состояния компенсации углеводного обмена по уровню HbA1c в динамике визитов показал отсутствие статистически значимых изменений до и после перенесенного COVID-19. Медиана HbA1c при СД1 до COVID-19 составила 9,0%, при обследовании в «Диамобиле» — 8,4% (p=0,537), при СД2 — 8,3 и 8,2% (p=0,341) (табл. 2), что может быть связано с интенсификацией сахароснижающей терапии (ССТ) в период инфекции, продолжавшейся в постковидный период. Так, при анализе ССТ у пациентов с СД2 в динамике отмечено, что после перенесенного COVID-19 увеличилась доля пациентов на двух- и трех- и более компонентной терапии на 4,3 и 1,6% соответственно, а также увеличилась доля пациентов на инсулинотерапии на 16% (рис. 2).

**Table:** Таблица 2. Сравнительный анализ динамики ИМТ, СКФ, HbA1c до и после подтвержденного COVID-19 у пациентов с СД1 и СД2 Данные представлены в виде медианы (Ме) и 1 и 3 квартиля [ Q1; Q3]. ИМТ — индекс массы тела, СКФ — скорость клубочковой фильтрации, HbA1c — гликированный гемоглобин.

Признак	До	После	p, Wilcoxon
Me [ Q1; Q3]	Me [ Q1; Q3]
СД 1 типа
ИМТ, кг/м²	25,71 [ 23,31; 27,92]	25,77 [ 22,77; 28,13]	0,552
СКФ, мл/мин/1,73м²	88,1 [ 70,8; 100,1]	62,0 [ 51,1; 74,3]	<0,001
HbA1c, %	9,0 [ 8,2; 9,6]	8,4 [ 7,4; 9,3]	0,537
СД 2 типа
ИМТ, кг/м²	29,16 [ 26,45; 31,57]	32,88 [ 29,76; 36,16]	<0,001
СКФ, мл/мин/1,73м²	74,7 [ 57,0; 84,3]	54,1 [ 47,8; 61,5]	<0,001
HbA1c, %	8,3 [ 7,0; 9,3]	8,2 [ 7,2; 9,4]	0,341

**Figure:**
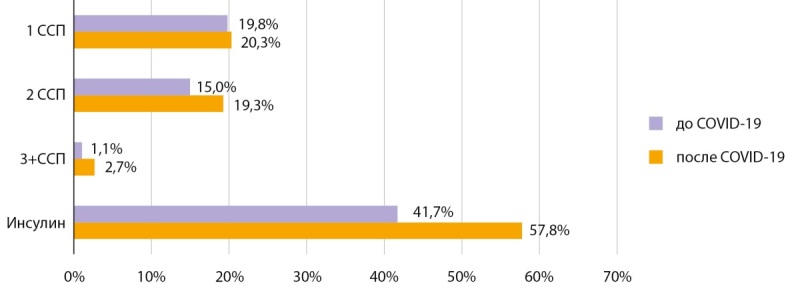
Рисунок 2. Структура сахароснижающей терапии в динамике до и после COVID-19 у пациентов с сахарным диабетом 2 типа. ССП — сахароснижающий препарат.

При анализе динамики лабораторных показателей до и после COVID-19 у пациентов с СД в «Диамобиле» были отмечены статистически значимые изменения следующих показателей: снижение СКФ после COVID-19 при СД1 с 88,1 до 62 мл/мин/1,73м² (p<0,001), при СД2 с 74,7 до 54,1 мл/мин/1,73м² (p<0,001) и повышение ИМТ у пациентов с СД2: с 29,16 кг/м² до 32,88 кг/м² (p<0,001).

При оценке доли (%) пациентов с наличием диабетических осложнений до и после COVID-19 отмечено увеличение частоты ком при СД1 в 1,5 раза с 8 до 12% и тяжелых гипогликемий в 3 раза с 2 до 6% (рис. 3), при СД2 частота ком не изменилась, но увеличилась регистрация тяжелых гипогликемий в 1,7 раза — с 1,6 до 2,7%. Данные тенденции могут быть связаны не только с фактом интенсификации ССТ, но и отражают трудности безопасного достижения целевого гликемического контроля на фоне вирусной инфекции, возможно, вследствие более выраженной вариабельности гликемии.

**Figure:**
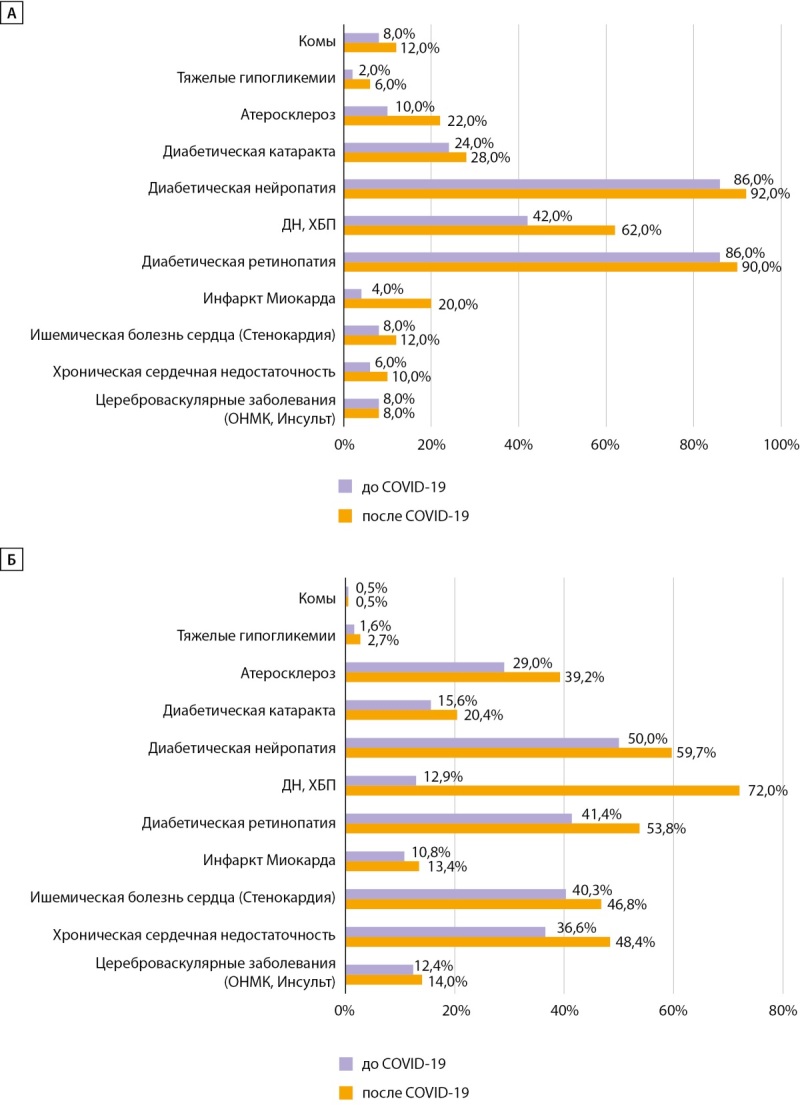
Рисунок 3. Структура диабетических осложнений до и после COVID-19 у пациентов с сахарным диабетом 1 типа (А) и 2 типа (Б). ДН — диабетическая нефропатия; ХБП — хроническая болезнь почек.

Частота хронических осложнений у пациентов с СД в постковидном периоде увеличилась кумулятивно по всем осложнениям: при СД1 — на 8,5%, при СД2 — на 13,2%. Частота кардиологических осложнений увеличилась при СД1 в диапазоне от 1,5 до 5 раз: так, инфаркт миокарда (ИМ) — в 5 раз, с 4 до 20%, ишемическая болезнь сердца (ИБС) — в 1,5 раза, с 8 до 12%, хроническая сердечная недостаточность (ХСН) — в 1,67 раза, с 6 до 10%, атеросклероз — в 2,2 раза, с 10 до 22%; при СД2 — в среднем в 1,3 раза: ИМ — с 10,8 до 13,4%, ИБС — с 40,3 до 46,8%, ХСН — с 36,6 до 48,4%, атеросклероз — в 1,35 раза, с 29 до 32,2% соответственно.

Наибольший прирост отмечался в отношении частоты хронической болезни почек (ХБП): при СД1 — в 1,5 раза (с 42 до 62%), при СД2 — в 5,6 раза (с 12,9 до 72%) (рис. 3).

При анализе пациентов по уровню СКФ до и после перенесенного COVID-19 отмечалось перераспределение в основном за счет существенного сокращения доли пациентов с СКФ≥60 мл/мин/1,73 м²: при СД1 — с 92 до 62%, при СД2 — с 68 до 28%, вследствие значимого прироста доли пациентов, развивших снижение функции почек, соответствующее развитию ХБП по критериям СКФ (<60 мл/мин/1,73 м²) суммарно ХБП С3а-5 стадий при СД1 возросло от исходных 8 до 38%, при СД2 — с 32 до 74%, преимущественное увеличение за счет 3а стадии при обоих типах СД. У пациентов с СД2 отмечалось более выраженное снижение функционального состояния почек, так, доля пациентов со значительным снижением СКФ <30мл/мин/1,73 м² возросла в 4 раза с 6 до 24% (рис. 4).

**Figure:**
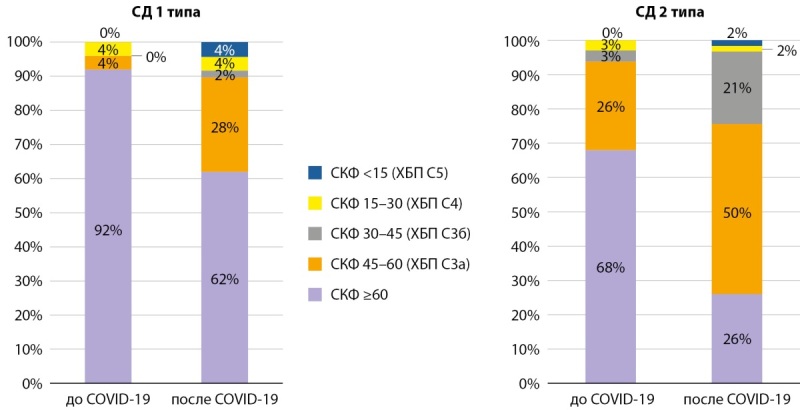
Рисунок 4. Распределение пациентов по скорости клубочковой фильтрации и стадиям ХБП до и после COVID-19 у пациентов с сахарным диабетом 1 и 2 типов. СКФ — скорость клубочковой фильтрации в мл/мин/1,73 м².

## ОБСУЖДЕНИЕ

В статье представлен комплексный анализ клинико-эпидемиологического обследования пациентов с СД в «Диамобиле» с оценкой динамики частоты диабетических осложнений и уровня компенсации углеводного обмена до и после перенесенного COVID-19.

Мировые данные по частоте встречаемости постковидного синдрома различны, распространенность, по данным литературы, варьирует от 10–20% до 30–70% [13][14]. Развитие постковидного синдрома чаще отмечается в тех же группах высокого риска летальности и тяжелого течения COVID-19. По данным различных публикаций, постCOVID-синдром имеют от 10 до 35% пациентов, не нуждающихся в госпитализации, независимо от сопутствующих заболеваний [15]. Среди пациентов, госпитализированных с тяжелым течением SARS-CoV-2-инфекции, частота постковидного синдрома достигает 80% [16].

Большое количество данных о постковидных нарушениях получено из публикаций на основе международного регистра COVID-19 «Анализ динамики Коморбидных заболеваний у пациентов, перенесших инфицирование SARS-CoV-2» (AКТИВ SARS-CoV-2), с российским участием [17]. Авторами сделано заключение, что полиморбидность, определенная наличием сопутствующих заболеваний, является фактором риска летальности при коронавирусной инфекции. В состав наиболее часто встречающихся комбинаций у пациентов с COVID-19 вошли сердечно-сосудистые заболевания, сахарный диабет и ожирение. Наличие таких комбинаций приводило к повышению риска летального исхода вследствие COVID более, чем в 4 раза [18], однако когорта пациентов с СД отдельно не изучалась.

В популяции пациентов с СД эпидемиология постковидного синдрома в настоящий момент изучена недостаточно [19][20]. Одной из причин является отсутствие точного определения постковидного синдрома и критериев его диагностики. В когорте пациентов с СД дополнительные трудности в верификации данного состояния вносят необходимость дифференциальной диагностики с развитием хронических диабетических осложнений, имеющих общие патогенетические механизмы развития, с выделением постковидного компонента. Вирусная инфекция COVID-19 при СД рассматривается в качестве мощного промотирующего фактора поражения сердечно-сосудистого русла в рамках развития хронических и острых диабетических осложнений, сопровождаемого повышением риска летальности. Это определяет необходимость комплексного подхода при обследовании пациентов в оценке полиорганной дисфункции, что чрезвычайно важно именно для пациентов с СД.

В основе механизмов развития постковидного синдрома рассматривают 3 вида повреждения органов и тканей: первое — это прямое влияние непосредственно самого вируса, второе — нарушения, опосредованные активизацией иммунной системы, третье — дисфункция различных органов и систем, как следствие тяжелого соматического заболевания [21].

Полиорганное повреждение при СД и COVID-19 имеет общую патофизиологическую основу, связанную с тканевым воспалением и эндотелиальной дисфункцией. Клетки эндотелия экспрессируют рецепторы к АСЕ2, которые являются лигандом для проникновения вируса SARS-CoV-2 [22]. Происходит прямое вирусное поражение сосудов с активацией провоспалительных и протромбогенных факторов и нарушением микроциркуляции, что приводит к поражению органов мишеней с развитием острых СС катастроф или хронической кардиоренальной недостаточности.

РАС является системой, модулирующей СС нарушения при любой патологии. На этапе начала пандемии предполагалось, что индивидуальные особенности РАС могут влиять на выраженность клинических проявлений COVID-19 и развитие постковидного синдрома [23]. Проведенные исследования показали: степень тяжести клинических проявлений COVID-19 коррелирует с уровнем воспалительных маркеров вне зависимости от генетического и функционального состояния РАС (активности плазменных компонентов РАС и полиморфизма гена ACE2), что указывает на ведущую роль системного воспаления в качестве фактора, определяющего тяжесть нарушений при COVID-19 [24].

Клинический опыт наблюдений в общей популяции позволил классифицировать 4 основных вида постковидных нарушений: 1) гипоксический синдром (дыхательная и кислородная недостаточность); 2) астенический синдром (общая слабость и низкая толерантность к физическим нагрузкам); 3) синдром психоневрологических нарушений (снижение настроения, депрессия, ухудшение когнитивных способностей, аносмия, нарушения сна); 4) гастроинтестинальные симптомы (диспепсия, дисбактериоз, повышение печеночных ферментов, искажение и снижение вкусовых ощущений) [25]. Следует отметить, что в данной градации не выделена группа кардиоренальных поражений, в то время как сердечно-сосудистые и почечные осложнения представляют наибольшую угрозу для прогноза пациентов.

В проведенном нами исследовании было показано, что при относительно стабильных показателях гликемического контроля до и после COVID-19 отмечено значимое снижение СКФ. Так, после перенесенного COVID-19 при СД2 частота всех сосудистых осложнений выросла на 13,2%, в то время как частота ХБП увеличилась на 59,1%, то есть возросла в 5,6 раза. Таким образом, почки можно рассматривать в качестве основного органа поражения вследствие COVID-19 у пациентов с СД.

В когортном исследовании 1733 пациентов, перенесших COVID-19, при исходных значениях более 90 мл/мин/73 м² у 13% пациентов спустя 6 месяцев было диагностировано снижение СКФ менее 90 мл/мин/73 м² (у 107 из 822) [26]. В других работах снижение СКФ в постковидном периоде отмечено у 12–22% пациентов [27].

Вовлеченность почек в патологический процесс при COVID-19 верифицирована при электронной микроскопии. Так, по данным исследования [28], в ткани почек при аутопсийном исследовании были выявлены вирусные включения в перитубулярном пространстве, эндотелиальных клетках петель капилляров клубочков и базальной мембране клубочка, то есть основных структурах почки, определяющих ее функциональную активность.

Существует гипотеза, что вследствие прямого токсического влияния вируса развивается серия краткосрочных острых почечных нарушений, которые реализуются в хроническое снижение СКФ [29]. Тяжелая острая почечная недостаточность (ОПН), требующая заместительной почечной терапии (ЗПТ), встречается у 5% госпитализированных пациентов с COVID-19, у пациентов, госпитализированных в отделение неотложной помощи, распространенность ОПН достигает 20–31% [30]. Морфологически снижение функции почек обусловлено развитием очагово-сегментарного гломерулосклероза, образованием тромбов в микро-циркуляторном русле почек и повреждением канальцев в острую фазу заболевания [31]. Нарушение почечной функции в постковидном периоде протекает по разному пути, что требует динамического наблюдения и тщательного мониторинга этих пациентов. Так, анализ 115 пациентов, госпитализированных в ОРИТ с ОПН, потребовавшей ЗПТ в острую фазу COVID-19, продемонстрировал очень высокую смертность в этой когорте (51% пациентов), при этом среди выживших большинство полностью восстановили почечную функцию к моменту выписки (84% больных) и 8% нуждались в продолжении диализа [32]. В то же время у пациентов без предшествующего анамнеза ОПН в острую фазу отмечалось снижение СКФ в течение года после перенесенной новой коронавирусной инфекции.

Таким образом, это еще раз подчеркивает важность состояния почечного функционала для жизненного прогноза пациентов и определяет необходимость изучения ренальных нарушений при COVID-19 и в постковидном периоде.

По мере накопления клинического материала о постковидных изменениях становится все более очевидна актуальность классификации этого коморбидного состояния, разработки алгоритма выделения основных групп риска и применения научно обоснованных подходов к ведению и лечению пациентов после COVID-19, особенно в группах риска, к которым относятся пациенты с СД.

Выполненный анализ обусловливает важность организации системы учета и мониторинга пациентов с СД с целью предупреждения рисков прогрессирования кардиоренальных осложнений и риска смертности после перенесенного COVID-19. Внедрение региональных программ мониторинга пациентов с СД с использованием мобильных лечебно-диагностических центров могло бы существенно повысить доступность специализированной помощи, в том числе в отдаленных и сельских районах, что имеет особую актуальность в условиях кадрового дефицита.

## Ограничения исследования

При обсуждении полученных результатов следует учитывать, что механизм развития хронических диабетических осложнений и патофизиологические аспекты постковидного синдрома имеют много общего и могут обоюдно усугублять и потенциировать развитие нарушений. Таким образом, четко дифференцировать постковидные изменения от старта хронического диабетического осложнения в данный период не всегда представляется возможным, что обусловливает определенные ограничения при интерпретации результатов нашего исследования. Разработанный дизайн с применением уникального инструмента клинико-эпидемиологического мониторинга посредством «Диамобиля», с проведением стандартизованной оценки состояния основных органов мишеней после перенесенного COVID-19, несколько уменьшает, но не исключает ограничения, поскольку показатели исходного визита фиксировались из регистра СД. То есть имело место влияние таких факторов, как проведение исследований разными врачами, в разных лабораториях, на разном оборудовании. Кроме того, существуют общие ограничения, связанные с дизайном наблюдательных исследований и отсутствием группы контроля.

## ЗАКЛЮЧЕНИЕ

В нашем исследовании установлено, что несмотря на стабильные показатели HbA1c и отсутствие значимого ухудшения гликемического контроля, в том числе вследствие интенсификации ССТ на фоне COVID-19, у пациентов с СД1 и СД2 в постковидном периоде наблюдается ухудшение функциональной способности почек (снижение СКФ) и увеличение частоты сердечно-сосудистых осложнений при обоих типах СД, что определяет ключевой спектр мероприятий для разработки мер профилактики. Проведение контрольных эпидемиологических исследований посредством «Диамобиля» предлагается в качестве инструмента оценки постковидных нарушений в когортных выборках пациентов для их своевременной диагностики в условиях первичного звена.

## ДОПОЛНИТЕЛЬНАЯ ИНФОРМАЦИЯ

Источники финансирования. Исследование выполнено при финансовом обеспечении государственного задания Минздрава России, НИОКТР № 122012100183-1.

Конфликт интересов. Авторы декларируют отсутствие явных и потенциальных конфликтов интересов, связанных с публикацией настоящей статьи.

Участие авторов. Викулова О.К., Серков А.А., Исаков М.А., Вагапова Г.Р., Валеева Ф.В., Трубицына Н.П., Мельникова О.Г., Александрова В.К., Смирнова Н.Б., Егорова Д.Н., Артемова Е.В., Сорокина К.В. — личное участие в выезде «Диамобиля». Викулова О.К., Железнякова А.В. — анализ данных и интерпретация результатов, написание текста статьи. Шестакова М.В., Дедов И.И., Мокрышева Н.Г. — одобрение финальной версии рукописи. Все авторы одобрили финальную версию статьи перед публикацией, выразили согласие нести ответственность за все аспекты работы, подразумевающую надлежащее изучение и решение вопросов, связанных с точностью или добросовестностью любой части работы.

Благодарности. Всем медицинским специалистам (врачам, медицинским сестрам, регистраторам данных), принимавшим участие в работе мобильного медицинского центра «Диамобиль» в Республике Татарстан. Авторы выражают благодарность врачам, медсестрам и другим медицинским специалистам, обеспечивавшим активный ввод сведений в базу данных регистра СД.
